# Vital Signs: Sodium Intake Among U.S. School-Aged Children — 2009–2010

**Published:** 2014-09-12

**Authors:** Mary E. Cogswell, Keming Yuan, Janelle P. Gunn, Cathleen Gillespie, Sarah Sliwa, Deborah A. Galuska, Jan Barrett, Jay Hirschman, Alanna J. Moshfegh, Donna Rhodes, Jaspreet Ahuja, Pamela Pehrsson, Robert Merritt, Barbara A. Bowman

**Affiliations:** 1 Division for Heart Disease and Stroke Prevention, National Center for Chronic Disease Prevention and Health Promotion, CDC; 2 Division of Population Health, National Center for Chronic Disease Prevention and Health Promotion, CDC; 3 Division of Nutrition, Physical Activity and Obesity, National Center for Chronic Disease Prevention and Health Promotion, CDC; 4 Child Nutrition Program, Food and Nutrition Service, U.S. Department of Agriculture; 5 Special Nutrition Research and Analysis Division, Food and Nutrition Service, U.S. Department of Agriculture; 6 Food Surveys Research Group; 7 Nutrient Data Laboratory, Agricultural Research Service, US Department of Agriculture

## Abstract

**Background:**

A national health objective is to reduce average U.S. sodium intake to 2,300 mg daily to help prevent high blood pressure, a major cause of heart disease and stroke. Identifying common contributors to sodium intake among children can help reduction efforts.

**Methods:**

Average sodium intake, sodium consumed per calorie, and proportions of sodium from food categories, place obtained, and eating occasion were estimated among 2,266 school-aged (6–18 years) participants in *What We Eat in America*, the dietary intake component of the National Health and Nutrition Examination Survey, 2009–2010.

**Results:**

U.S. school-aged children consumed an estimated 3,279 mg of sodium daily with the highest total intake (3,672 mg/d) and intake per 1,000 kcal (1,681 mg) among high school–aged children. Forty-three percent of sodium came from 10 food categories: pizza, bread and rolls, cold cuts/cured meats, savory snacks, sandwiches, cheese, chicken patties/nuggets/tenders, pasta mixed dishes, Mexican mixed dishes, and soups. Sixty-five percent of sodium intake came from store foods, 13% from fast food/pizza restaurants, 5% from other restaurants, and 9% from school cafeteria foods. Among children aged 14–18 years, 16% of total sodium intake came from fast food/pizza restaurants versus 11% among those aged 6–10 years or 11–13 years (p<0.05). Among children who consumed a school meal on the day assessed, 26% of sodium intake came from school cafeteria foods. Thirty-nine percent of sodium was consumed at dinner, followed by lunch (29%), snacks (16%), and breakfast (15%).

**Implications for Public Health Practice:**

Sodium intake among school-aged children is much higher than recommended. Multiple food categories, venues, meals, and snacks contribute to sodium intake among school-aged children supporting the importance of populationwide strategies to reduce sodium intake. New national nutrition standards are projected to reduce the sodium content of school meals by approximately 25%–50% by 2022. Based on this analysis, if there is no replacement from other sources, sodium intake among U.S. school-aged children will be reduced by an average of about 75–150 mg per day and about 220–440 mg on days children consume school meals.

## Introduction

A *Healthy People 2020* (HP2020) objective is to reduce average sodium intake in the U.S. population aged ≥2 years to decrease the risk of high blood pressure (hypertension), a major cause of heart disease and stroke (1). The target is 2,300 mg daily, a decrease of approximately 40% from current intake.* A 40% reduction in U.S. sodium intake is projected to save 280,000 to 500,000 lives over 10 years (2). Although hypertension, heart disease, and stroke are more common among adults, their origins can be in childhood: an estimated one in six U.S. children aged 8–17 years have pre–high blood pressure or high blood pressure (3). Children with higher blood pressure are more likely to develop hypertension as adults, making early prevention imperative (4–8).†

Average sodium consumption among U.S. children does not meet HP2020 targets. Over 90% of U.S. school-aged children and adolescents consume too much sodium (9) relative to the *Dietary Guidelines for Americans* and the Institute of Medicine (IOM) upper intake levels.§ Average daily sodium consumption declined slightly over the past decade among younger (aged ≤13 years) school-aged children, but not among adolescents, and not in terms of sodium consumed per calorie (9). It is estimated that more than three fourths of sodium intake comes from commercially processed packaged and restaurant foods (10). To reduce U.S. sodium intake, the primary strategy recommended by IOM is reductions in the sodium content of commercially processed and restaurant foods (10). Additionally, new national nutrition standards for school meals and other foods sold in schools might help reduce sodium intake among children who consume these foods.¶

Identifying the major food sources among U.S. school-aged children can aid in developing strategies for reducing sodium consumption in this population. This report describes mean sodium intake, sodium density (defined as mg of sodium per 1,000 kcal), and the food categories, places obtained (e.g., restaurant), and eating occasions contributing to sodium intake among U.S. children aged 6–18 years during 2009–2010.

## Methods

The National Health and Nutrition Examination Survey is an ongoing, nationally representative, multistage, stratified survey of the U.S. noninstitutionalized population.** During 2009–2010, 2,375 children aged 6–18 years were interviewed and examined (about 85%–87% of those screened). Of these, 2,266 completed an initial, in-person, 24-hour dietary recall as part of *What We Eat in America* (WWEIA), the dietary intake component of the National Health and Nutrition Examination Survey. Details on the 24-hour dietary recall and food and nutrient analysis have been published.††,§§,¶¶ Estimates excluded sodium from salt added at the table (estimated to be about 5% of intake) (10).

To identify foods contributing to sodium consumption, foods similar in use and nutrient content were grouped together using WWEIA food categories for 2009–2010.*** Food categories were ranked based on their percentage contribution to total sodium intake among U.S. children aged 6–18 years, calculated as the sum of the sodium from foods consumed from a specific category, divided by the sum of sodium consumed from all foods for all persons, and multiplied by 100. Food categories contributing to sodium intake were examined by age, sex, race/ethnicity, family income, and weight status. Age groups correspond to enrollment in elementary (6–10 years), middle (11–13 years), and high school (14–18 years). Family income corresponds with eligibility to receive free (≤130% of poverty) or reduced-price (>130%–185%) school meals. Weight status was determined by body mass index (weight [kg]/[height {m}]^2^) for age and sex percentile in reference to the 2000 CDC growth charts.†††

To determine how foods obtained from a specific setting (i.e., school cafeteria) contributed to total sodium intake among children who consumed a school meal, responses of 568 children (with a weekday dietary recall) who met the following criteria were analyzed separately: “now attending school” and “usually get a complete school lunch” or a “school breakfast” five times a week.

Wald F tests were used to examine whether means differed among subgroups, and t-tests were used to examine differences between age groups (e.g., ages 6–10 years compared with 14–18 years) in proportions of sodium consumed and mean sodium density from different places (e.g., stores and restaurants). Statistical software accounting for the complex survey design was used for all analyses. Each participant was assigned a numerical sample weight equivalent to the number of children in the population represented by that person. Sample weights for NHANES participants incorporate adjustments for unequal selection probabilities by specific age, sex, or race/ethnicities and certain types of nonparticipation or nonresponse. The numerical sample weights for the (day 1) 24-hour dietary recall were used for all analyses.

## Results

Mean daily sodium consumption was 3,279 mg, and mean sodium density was 1,638 mg sodium per 1,000 kcal. Total sodium intake was highest among high school–aged children and among males (Table 1), lowest among children (with family income ≤185% of the federal poverty level) who qualified for reduced-price school meals (Table 2), and did not vary by race/ethnicity (Table 1) or weight status (Table 2). Sodium density was highest among high school–aged children and varied by age group (p=0.01), but not by other examined variables (p≥0.05).

Approximately 43% of sodium among U.S. children aged 6–18 years was consumed from foods in the following 10 categories: 1) pizza; 2) yeast bread and rolls; 3) cold cuts/cured meats; 4) savory snacks (e.g., chips, pretzels, and popcorn); 5) sandwiches§§§; 6) cheese; 7) chicken patties, nuggets, and tenders; 8) pasta mixed dishes (including spaghetti with meat sauce but excluding macaroni and cheese); 9) Mexican-mixed dishes¶¶¶; and 10) soups (Tables 1 and 2). The five leading food categories consistently appeared among the top 10 ranked categories among the subgroups examined.

Among participants, 65.1% of sodium consumed came from foods (or ingredients) obtained from a store (e.g., supermarket, warehouse store); 13.0% from fast food/pizza restaurants, 4.9% from other restaurants, 9.1% from the school cafeteria, and 7.4% from other sources (Table 3). Among high school–aged (14–18 years) participants, a higher proportion of total sodium intake came from fast food/pizza restaurants (15.5%) compared with children aged 6–10 years (10.9%) and 11–13 years (10.8%), p<0.05). Among elementary school–aged versus middle and high school–aged children, a higher proportion came from school cafeteria foods (11.7% versus 8.9% and 7.4%, respectively, p<0.05). Among participants who consumed a school meal on the day of recall, 26.0% of sodium came from school cafeteria foods; this proportion did not vary by age group.

Overall, the mean sodium density (1,843 mg/1,000 kcal) was highest in fast food/pizza restaurants foods. Among high school–aged, compared with elementary and middle school–aged children, the mean sodium density from school cafeteria foods was greater (1,828 versus 1,528 and 1,617, respectively, p<0.05) and did not appear to differ from mean sodium density from fast food/pizza restaurant foods among high school–aged children (1,817).

**Key Points**

- A national health objective for 2020 is to reduce average daily sodium intake by about 40% to 2,300 mg, projected to save 280,000 to 500,000 lives over 10 years.
- Total sodium intake was 3,279 mg, higher among high school–aged children than other children. The amount of sodium consumed per calorie also was higher among high school–aged versus younger children, but otherwise did not vary by group.
- Although foods from grocery stores contribute the majority of sodium intake, foods from fast-food/pizza restaurants continue to contribute higher amounts of sodium per calorie, and contribute higher proportions of total sodium intake among high school–aged versus younger children.
- Among children aged 6–18 years, 9% of total sodium intake came from school cafeterias; among 568 children who consumed a school meal on their 24-hour dietary recall day, 26% of total sodium intake came from school cafeterias.
- Approximately 43% of sodium was consumed from foods in the following 10 categories: pizza; yeast bread and rolls; cold cuts/cured meats; savory snacks (e.g., chips and pretzels); sandwiches like cheeseburgers; cheese; chicken patties, nuggets, and tenders; pasta mixed dishes (including spaghetti with meat sauce but excluding macaroni and cheese); Mexican-mixed dishes (e.g., burritos and tacos); and soups.
- Additional information is available at http://www.cdc.gov/vitalsigns.

When examined by eating occasion, 39.2% of sodium intake occurred at dinner; 29.5% lunch; 16.4% snacks; and 14.9% breakfast (Table 4). Among high school–aged compared with younger children, a lower proportion of total sodium intake came from breakfast (p<0.05). In addition to foods obtained from a store, which contributed 11.3% to 26.0% of total sodium intake across eating occasions on a typical day, foods from school cafeterias at lunch contributed 7.2% to sodium intake and from fast/food pizza restaurants at dinner, 6.6%. This pattern differed little by age, except among high school–aged children, for whom foods from fast/food pizza restaurants consumed at lunch contributed the same as foods from school cafeterias.

## Conclusions and Comments

U.S. school-aged children, on average, consume sodium in excess of recommended levels regardless of age, sex, race-ethnicity, income or weight status. The top 10 food categories contributed >40% of total sodium intake and, in general, varied little among population subgroups. Although foods from grocery stores contribute the majority of sodium intake, fast-food/pizza restaurant foods have the highest sodium density (11). Among high school–aged compared with younger children, school cafeteria foods also contribute high sodium density. Results support the need for sodium reduction across multiple foods, venues, and eating occasions.

These are the most current analyses regarding contributors to population sodium consumption among U.S. school-aged children. As in 2007–2008 analyses (11), results indicate mean sodium intake is higher among older age groups and reaching adult levels among adolescents aged 14–18 years. Although this difference appears related to greater energy requirements and intake, high school–aged children also consumed more sodium per calorie versus younger age groups, suggesting the greater sodium intake among this age group was related to both higher sodium content and the amount of foods consumed. The higher the sodium density, the less effective caloric reduction could be as a single strategy to reduce sodium intake. As several commonly consumed foods (e.g., pizza and yeast breads/rolls/buns) are leading contributors to children’s intake of both sodium and energy, strategies to reduce consumption of sodium-dense foods and/or replace them with lower sodium versions of these foods or potassium rich foods (e.g., fruits and vegetables) might advance efforts to prevent higher blood pressure and leverage ongoing efforts to prevent and reduce childhood obesity.

Children who qualified for reduced price school meals had the lowest average sodium intake, but sodium density and types of food contributing to sodium intake did not differ significantly. Given that sodium and energy intake are highly correlated, this finding is likely related to energy intake.

Among children who consume school meals, mean energy intakes from foods obtained from the cafeteria (470–542 kcal) were consistent with 2004–2005 School Nutrition and Dietary Assessment data (517–546 kcal) (12). Among high school–aged children, the high sodium density from foods from school cafeterias might be related to greater availability of competitive foods (e.g., a la carte options) sold in schools separately from the National School Lunch Program or School Breakfast Programs. Among younger children, the slightly greater contribution (higher proportion) of school cafeteria foods to overall sodium intake might be related to higher participation rates among elementary school versus older children in school meal programs.****

The findings in this report are subject to at least seven limitations. First, institutionalized populations were excluded, and results might be influenced by nonresponse bias unaccounted for by the sample weights. Second, the food code used in WWEIA to estimate the nutrient content of a specific food (e.g., pizza) was the same across venues (e.g., schools and stores), but the foods served in these settings might vary in sodium content. Third, ranking is greatly influenced by categorization method (e.g. sandwiches do not include those coded as separate components, such as bread).†††† Fourth, dietary recall data are subject to reporting error. Fifth, comparisons do not control for differences in other characteristics across groups. Sixth, the 568 children who consumed school meals might not represent all children participating in the National School Lunch Program or School Breakfast Program. Children who skipped meals and/or did not consume a complete school lunch or breakfast are included in the analyses (13). Questions about foods obtained from school cafeterias do not specify whether the food was obtained from the National School Lunch Program or School Breakfast Program or other foods sold in the cafeteria. Finally, statistical power was limited in the ability to examine results from smaller subgroups.

Commonly consumed foods, such as pizza, bread, cold cuts, savory snacks, and sandwiches contributed to excess sodium intake among school-aged children. These findings are consistent across population subgroups, reinforcing the IOM recommendations to set phased targets to reduce the sodium content of U.S. commercially processed foods (10) to achieve national health objectives. In the United Kingdom, use of this strategy for commercially processed packaged foods was associated with a 15% decrease in mean sodium intake over 7 years (14). IOM recommends also setting targets for commercially processed restaurant foods to further reduce sodium intake (10). Complementing this strategy, national nutrition standards for school breakfasts and lunches and almost all foods sold in U.S. schools set phased targets for sodium content starting with the 2014–2015 school year, and evidence suggests that reducing the sodium content of these foods is achievable (15). These phased targets are estimated to result in a 25%–50% sodium reduction in school meals by 2022. Considering sodium from school cafeteria foods contributes about 300 mg daily to overall sodium intake among U.S. school aged children, and about 870 mg daily on days children consume school meals, this measure could reduce sodium intake by 75–150 mg per day overall, and about 220–440 mg on days school meals are consumed, if there is no replacement from other sources.

Meta-analyses of studies of diverse groups of children have found that lowering sodium intake reduces average blood pressure; a 42% sodium reduction in children reduces average blood pressure by 0.6–1.8 (systolic)/0.7–1.9 (diastolic) mm Hg (7,8). A 2 mm Hg reduction, if maintained into adulthood, could translate into a large reduction in heart attacks and strokes and subsequent mortality (2,16). Given the relationship between sodium reduction and high blood pressure, sodium reduction is an important part of the strategy to help prevent 1 million heart attacks and strokes by 2017.§§§§

## Figures and Tables

**TABLE 1:** Ranked proportions of sodium consumed by children aged 6–18 years,* by selected food categories, age groups, sex, and race/ethnicity — National Health and Nutrition Examination Survey, United States, 2009–2010

		Age group (yrs)				Sex		Race/Ethnicity		
				
		6–18 overall	6–10	11–13	14–18	Male	Female	Hispanic	Black, non-Hispanic	White, non-Hispanic
										
Rank†	Food category§	% (SE)	% (SE)	% (SE)	% (SE)	% (SE)	% (SE)	% (SE)	% (SE)	% (SE)
1	Pizza	8.4 (0.9)	7.4 (1.0)	8.5 (1.0)	9.0 (1.9)	9.3 (1.3)	7.2 (0.8)	8.2 (0.6)	9.2 (1.5)	8.4 (1.5)
2	Yeast breads/rolls/buns¶	5.8 (0.3)	6.3 (0.3)	6.2 (0.8)	5.2 (0.4)	6.2 (0.4)	5.4 (0.4)	5.3 (0.4)	5.1 (0.4)	6.4 (0.5)
3	Cold cuts/cured meats	4.7 (0.5)	3.4 (0.4)	5.9 (1.0)	5.2 (0.7)	5.3 (0.7)	4.1 (0.7)	3.5 (0.6)	3.4 (1.0)	5.7 (0.9)
4	Savory snacks**	4.3 (0.3)	4.2 (0.3)	4.8 (1.0)	4.2 (0.3)	4.6 (0.4)	4.0 (0.2)	4.3 (0.4)	4.4 (0.5)	4.4 (0.4)
5	Sandwiches (single code)††	4.0 (0.4)	3.7 (0.5)	4.0 (0.7)	4.4 (0.5)	4.3 (0.4)	3.7 (0.5)	4.3 (0.9)	3.9 (0.9)	4.0 (0.3)
6	Cheese	3.6 (0.2)	3.2 (0.2)	3.7 (0.4)	3.9 (0.3)	3.1 (0.3)	4.2 (0.3)	3.6 (0.3)	3.4 (0.4)	3.8 (0.3)
7	Chicken patties/nuggets/etc.§§	3.3 (0.6)	4.0 (0.6)	3.2 (0.8)	3.0 (0.9)	2.9 (0.5)	3.9 (0.9)	2.6 (0.3)	4.4 (0.6)	3.6 (1.0)
8	Pasta mixed dishes¶¶	3.1 (0.4)	3.1 (0.4)	3.5 (1.3)	2.9 (0.5)	2.5 (0.4)	3.9 (0.7)	2.6 (0.4)	3.6 (0.9)	2.8 (0.3)
9	Mexican mixed dishes***	3.0 (0.4)	2.1 (0.5)	2.5 (0.6)	3.8 (0.9)	3.2 (0.6)	2.7 (0.5)	4.8 (0.7)	2.4 (0.8)	2.6 (0.6)
10	Soups	2.9 (0.2)	4.1 (0.5)	2.2 (0.6)	2.4 (0.4)	3.0 (0.4)	2.9 (0.3)	5.0 (0.5)	2.9 (0.4)	1.9 (0.3)
**Mean daily sodium consumed**										
As measured in mg		**3,279**	**2,903**	**3,194**	**3,672** †††	**3,626**	**2,943** †††	**3,125**	**3,202**	**3,278**
(SE)		**(84)**	**(46)**	**(103)**	**(157)**	**(112)**	**(74)**	**(61)**	**(115)**	**(111)**
**Mean daily energy consumed**										
As measured in kcal		**2,017**	**1,845**	**1,983**	**2,195** †††	**2,220**	**1,821** †††	**1,963**	**2,027**	**2,023**
(SE)		**(36)**	**(20)**	**(51)**	**(64)**	**(55)**	**(30)**	**(24)**	**(71)**	**(47)**
**Mean daily sodium density**										
As measured in mg/1,000 kcal		**1,638**	**1,586**	**1,646**	**1,681** §§§	**1,637**	**1,639**	**1,611**	**1,586**	**1,636**
(SE)		**(15)**	**(11)**	**(25)**	**(29)**	**(14)**	**(21)**	**(20)**	**(24)**	**(21)**
**Unweighted no. of participants in sample**		**2,266** ¶¶¶	**943**	**509**	**814**	**1,170**	**1,096**	**901**	**461**	**748**

**Abbreviation:** SE = standard error.

* The proportion (%) of sodium consumed is defined as the sum of the amount of sodium consumed from each specific food category for all participants divided by the sum of sodium consumed from all food categories for all participants multiplied by 100. All estimates use 24-hour dietary recall, take into account the complex sampling design, and use dietary day 1 sample weights to account for nonresponse and weekend/weekday recalls.

† Rank based on population proportions of sodium consumed for the overall U.S. population aged 6–18 years. Columns for other groups are ordered by this ranking.

§ Additional information regarding food categorization is available at the *What We Eat in America* website, http://www.ars.usda.gov/services/docs.htm?docid=18349. Food categories contributing ≥3% to overall sodium consumption within specific sociodemographic groups but not listed among the top 10 contributors were as follows: Children aged 6–10 years, milk (unflavored), whole, reduced, low, and no-fat (3.4%) and frankfurters and sausages (3.3%); children aged 11–13 years, pancakes, waffles, and French toast (3.1%), chicken, whole pieces, and other poultry (3.0%); children aged 14–18 years, chicken whole pieces and other poultry (3.0%); Hispanic children, chicken whole pieces and other poultry (3.6%), tortillas (3.4%); non-Hispanic black children (chicken whole pieces and other poultry (5.2%), frankfurters and sausages (3.4%), tomato-based condiments (3.3 %); non-Hispanic white children, milk (unflavored), whole, reduced, low, and no-fat (3.2%).

¶ Excludes bagels and English muffins.

** Tortilla, corn, and other chips/pretzels/snack mix/potato chips/popcorn.

†† Sandwiches as identified by a single *What We Eat in America* food code, chicken or turkey sandwiches/burgers/egg/breakfast sandwiches/other sandwiches (e.g., corn dog).

§§ Includes chicken tenders. Excludes chicken whole pieces and turkey, duck, and other poultry.

¶¶ e.g., spaghetti with meat sauce or meat balls (excludes macaroni and cheese).

*** Burritos and tacos/nachos/other Mexican mixed dishes.

††† Statistically significant differences in mean sodium intake across subgroups, determined by the Wald F test (p<0.001).

§§§ Statistically significant differences in mean sodium intake per 1,000 kcal across subgroups, determined by the Wald F test (p=0.01).

¶¶¶ Includes other race/ethnicities not shown separately.

**TABLE 2:** Ranked proportions of sodium consumed by children aged 6–18 years,* by selected food categories, household income status, and weight status — National Health and Nutrition Examination Survey, United States, 2009–2010

		Household income relative to federal poverty level			Weight status†	
			
		≤130%	>130%–185%	>185%	Normal	Overweight/Obese
						
Rank§	Food category¶	% (SE)	% (SE)	% (SE)	% (SE)	% (SE)
1	Pizza	8.5 (1.3)	9.4 (2.3)	8.5 (1.4)	8.5 (1.2)	8.1 (1.0)
2	Yeast breads/rolls/buns**	5.6 (0.5)	5.5 (0.4)	6.0 (0.5)	6.0 (0.4)	5.4 (0.3)
3	Cold cuts/cured meats	4.6 (0.6)	2.4 (0.7)	5.5 (0.7)	5.3 (0.7)	3.9 (0.4)
4	Savory snacks††	3.9 (0.3)	5.7 (0.6)	3.8 (0.3)	4.2 (0.4)	4.5 (0.3)
5	Sandwiches (single code)§§	4.6 (0.7)	5.1 (1.1)	3.8 (0.4)	3.3 (0.4)	5.4 (0.8)
6	Cheese	3.9 (0.4)	3.2 (0.5)	3.6 (0.3)	3.7 (0.3)	3.4 (0.3)
7	Chicken patties/nuggets/tenders¶¶	3.5 (0.6)	3.7 (0.8)	3.5 (0.8)	3.3 (0.9)	3.8 (0.5)
8	Pasta mixed dishes***	3.2 (0.7)	2.7 (0.6)	3.2 (0.6)	3.3 (0.7)	2.9 (0.4)
9	Mexican mixed dishes†††	2.9 (0.8)	3.7 (1.1)	2.9 (0.7)	3.3 (0.5)	2.2 (0.3)
10	Soups	3.2 (0.3)	3.9 (1.0)	2.4 (0.3)	2.6 (0.4)	3.4 (0.6)
**Mean daily sodium consumed**						
As measured in mg		**3,316** §§§	**2,879**	**3,320**	**3,280**	**3,270**
(SE)		**(75)**	**(117)**	**(136)**	**(100)**	**(107)**
**Mean daily energy consumed**						
As measured in kcal		**2,007**	**1,881**	**2030**	**2,030**	**1,992**
(SE)		**(38)**	**(82)**	**(57)**	**(45)**	**(49)**
**Mean daily sodium density**						
As measured in mg/1,000 kcal		**1,667**	**1,562**	**1,645**	**1,629**	**1,654**
(SE)		**(17)**	**(41)**	**(21)**	**(17)**	**(27)**
**Unweighted no. of participants in sample**		**931**	**285**	**859**	**1352**	**834**

**Abbreviation:** SE = standard error.

* The proportion (%) of sodium consumed is defined as the sum of the amount of sodium consumed from each specific food category for all participants divided by the sum of sodium consumed from all food categories for all participants multiplied by 100. All estimates use 24-hour dietary recall, take into account the complex sampling design, and use dietary day 1 sample weights to account for nonresponse and weekend/weekday recalls.

† Normal was defined as a body mass index (BMI) for age and sex between the 5th and 85th percentiles. Overweight/obese was defined as a BMI for age and sex ≥85th percentile, based on specific reference values from the 2000 CDC growth charts.

§ Rank based on proportions of sodium consumed for the overall U.S. population aged 6–18 years. Columns for other groups are ordered by this ranking.

¶ Additional information regarding food categorization is available at the *What We Eat in America* website, http://www.ars.usda.gov/services/docs.htm?docid=18349. Food categories contributing ≥3% to overall sodium consumption within specific sociodemographic and weight status groups but not listed among the top 10 contributors overall were as follows: children with family income ≤130% of the federal poverty level, tomato-based condiments (3.5%), chicken whole pieces and other poultry (3.5%); family income >130%–185% of the poverty level, frankfurters and sausages (3.0%), dips, gravies, and other sauces (3.0%); overweight/obese children, tomato-based condiments (3.5%), chicken whole pieces and other poultry (3.1%).

** Yeast breads/rolls and buns, excludes bagels and English muffins.

†† Tortilla, corn, and other chips/pretzels/snack mix/potato chips/popcorn.

§§ Sandwiches as identified by a single *What We Eat in America* code, chicken or turkey sandwiches/burgers/egg/breakfast sandwiches/other sandwiches (e.g., corn dog).

¶¶ Excludes chicken whole pieces and turkey, duck, and other poultry.

*** e.g., spaghetti with meat sauce or meat balls (excludes macaroni and cheese).

††† Burritos and tacos/nachos/other Mexican mixed dishes.

§§§ Statistically significant differences in mean sodium intake across subgroups, determined by the Wald F test, p=0.01.

**TABLE 3:** Ranked proportions of sodium consumed by children aged 6–18 years* and mean sodium, energy, and sodium density intake,† by place obtained§ and age group among all participants and among those who consumed a school lunch or breakfast on any given day¶ *—* National Health and Nutrition Examination Survey, United States, 2009–2010

	Place obtained									
	
	Store		Restaurant with fast food/Pizza		Restaurant with waitstaff		Cafeteria at school		Other	
					
Participants/Age groups (yrs)	Value	(SE)	Value	(SE)	Value	(SE)	Value	(SE)	Value	(SE)
**All participants**										
**6–18 (Overall, N = 2,266)**										
Proportion of sodium %	65.1	(1.4)	13.0	(0.7)	4.9	(0.6)	9.1	(1.3)	7.8	(0.6)
Mean sodium in mg	2,135	(52)	426	(26)	162	(22)	299	(47)	257	(17)
Mean energy in kcal	1,346	(24)	231	(12)	92	(12)	179	(24)	169	(11)
Mean sodium density, mg/1,000 kcal	1,558	(18)	1,843	(52)	1,818	(45)	1,636	(50)	1,364	(56)
**6–10 (n = 943)**										
Proportion of sodium %	64.3	(2.1)	10.9	(1.1)	5.4	(1.1)	11.7	(1.4)**	7.6	(1.1)
Mean sodium in mg	1,868	(68)	318	(35)	157	(32)	340	(39)	221	(31)
Mean energy in kcal	1,206	(37)	171	(18)	86	(17)	217	(23)	165	(20)
Mean sodium density, mg/1,000 kcal	1,531	(21)	1,863	(67)	1,915	(81)	1,528	(42)	1,134	(43)***
**11–13 (n = 509)**										
Proportion of sodium %	67.6	(1.8)	10.8	(1.0)	3.8	(0.9)	8.9	(1.6)	9.0	(1.0)
Mean sodium in mg	2,160	(72)	345	(36)	120	(32)	283	(53)	286	(29)
Mean energy in kcal	1,354	(36)	197	(19)	76	(24)	172	(28)	185	(19)
Mean sodium density, mg/1,000 kcal	1,592	(16)††	1,873	(147)	1,618	(157)	1,617	(95)	1,691	(218)
**14–18 (n = 814)**										
Proportion of sodium %	64.5	(2.1)	15.5	(1.4)§§	5.2	(0.6)	7.4	(1.8)	7.5	(1.2)
Mean sodium in mg	2,367	(110)	569	(58)	190	(24)	271	(72)	274	(42)
Mean energy in kcal	1,471	(44)	305	(25)	107	(14)	147	(36)	165	(20)
Mean sodium density, mg/1,000 kcal	1,564	(46)	1,817	(76)	1,802	(78)	1,828	(63)¶¶	1,396	(98)
**Participants who consumed a school meal on their 24-hour dietary recall day**										
**6–18 (n = 568)**										
Proportion of sodium %	56.8	(2.2)	8.9	(1.1)	3.0	(0.7)	26.0	(2.2)	5.3	(1.4)
Mean sodium in mg	1,902	(109)	297	(39)	102	(26)	871	(89)	177	(48)
Mean energy in kcal	1,223	(48)	164	(20)	56	(12)	507	(47)	102	(22)
Mean sodium density, mg/1,000 kcal	1,589	(57)	1,800	(94)	1,945	(162)	1,705	(38)	1,619	(241)
**6–10 (n = 228)**										
Proportion of sodium %	55.3	(3.7)	6.4	(1.0)	—†††	—	29.0	(2.0)	4.4	(0.8)
Mean sodium in mg	1,702	(115)	196	(31)	—	—	892	(62)	136	(23)
Mean energy in kcal	1,059	(64)	111	(19)	—	—	542	(40)	87	(18)
Mean sodium density, mg/1,000 kcal	1,588	(69)	1,883	(110)	—	—	1,636	(51)	1,472	(156)
**11–13 (n = 157)**										
Proportion of sodium %	57.6	(2.2)	8.4	(2.1)	—	—	26.9	(2.4)	—	—
Mean sodium in mg	1,790	(113)	260	(70)	—	—	838	(70)	—	—
Mean energy in kcal	1,195	(66)	137	(34)	—	—	514	(38)	—	—
Mean sodium density, mg/1,000 kcal	1,516	(59)	1,901	(107)	—	—	1,632	(73)	—	—
**14–18 (n = 183)**										
Proportion of sodium %	57.6	(4.4)	11.1	(2.1)	2.2	(0.5)	23.2	(4.1)	—	—
Mean sodium in mg	2,161	(234)	416	(87)	84	(21)	870	(195)	—	—
Mean energy in kcal	1,396	(118)	231	(44)	41	(12)	470	(97)	—	—
Mean sodium density, mg/1,000 kcal	1,635	(131)	1,688	(155)	2,161	(156)§§§	1,854	(53)¶¶	—	—

**Abbreviation:** SE = standard error.

* The proportion (%) of sodium consumed is defined as the sum of the amount of sodium consumed from each specific food category for all participants divided by the sum of sodium consumed from all food categories for all participants multiplied by 100. All estimates use 24-hour dietary recall, take into account the complex sampling design, and use dietary day 1 sample weights to account for nonresponse and weekend/weekday recalls.

† A measure that accounts for differences in the amount of calories consumed from foods obtained from each source, defined as mg of sodium/1,000 kcal.

§ Place obtained was analyzed from responses to the question, “Where did you get this (most of the ingredients for this) [food name]?” Sources other than those shown were combined under “other” and included “from someone else/gift” (4.9% population proportion), and 19 other sources (e.g., vending machine), including “missing,” “do not know,” and “other/specify” (<1%).

¶ Analyzed separately were the responses of 568 children (with a weekday dietary recall) who met the following criteria: “now attending school” and “usually get a complete school lunch” or a “school breakfast” five times a week.

** Differences in population proportions compared with children aged 11–13 years and 14–18 years, T-tests, p<0.05.

†† Statistically significant difference in sodium density compared with children aged 6–10 years, by t-tests, p=0.02.

§§ Statistically significant difference in population proportions compared with children aged 6–10 years and 11–13 years, by t-tests, p<0.05.

¶¶ Statistically significant difference in mean sodium consumed per 1,000 kcal compared with children aged 6–10 years and 11–13 years, by t-tests, p<0.05.

*** Statistically significant difference in mean sodium consumed per 1,000 kcal compared with children aged 11–13 years and 14–18 years, by t-tests, p<0.05.

††† Estimates not reported, data are statistically unreliable, relative standard error ≥30%.

§§§ Statistically significant difference in mean sodium consumed per 1,000 kcal compared with children aged 11–13 years, by t-tests, p<0.05.

**TABLE 4:** Proportion* of sodium consumed from each eating occasion† and place obtained§ among children aged 6–18 years and by age group — National Health and Nutrition Examination Survey, United States, 2009–2010

		Eating occasion							
		
		Breakfast		Lunch		Dinner		Snack	
					
Age group (yrs)	Place obtained	%	(SE)	%	(SE)	%	(SE)	%	(SE)
**6–18 (N = 2,266)**									
	All	14.9	(0.6)	29.5	(1.0)	39.2	(1.1)	16.4	(0.6)
	Store	11.3	(0.4)	14.9	(1.0)	26.0	(1.1)	12.9	(0.5)
	Restaurants with fast food/Pizza	—¶		4.2	(0.5)	6.6	(0.5)	1.2	(0.2)
	Restaurant with waitstaff	—		1.3	(0.3)	3.0	(0.3)	—	
	School cafeteria	1.4	(0.3)	7.2	(1.3)	—		—	
	Other	—		1.9	(0.2)	3.4	(0.3)	1.9	(0.2)
**6–10 (n = 943)**									
	All	17.1	(0.6)	28.8	(0.8)	38.2	(1.4)	15.9	(0.6)
	Store	12.9	(0.6)	13.4	(1.2)	26.0	(1.4)	12.0	(0.6)
	Restaurants with fast food/Pizza	—		3.1	(0.5)	6.0	(0.5)	1.0	(0.3)
	Restaurant with waitstaff	—		2.0	(0.6)	3.0	(0.6)	—	
	School cafeteria	2.1	(0.3)	9.0	(1.2)	—		—	
	Other	—		1.3	(0.3)	3.0	(0.8)	2.4	(0.4)
**11–13 (n = 509)**									
	All food	15.8	(0.6)	26.4	(2.0)	40.3	(2.0)	17.5	(1.6)
	Store	12.4	(0.8)	13.8	(1.4)	27.1	(2.4)	14.3	(1.4)
	Restaurants with fast food/Pizza	—		2.3	(0.5)	6.9	(1.0)	—	
	Restaurant with waitstaff	—		—		2.0	(0.5)	—	
	School cafeteria	1.1	(0.4)	7.0	(1.5)	—		—	
	Other	—		2.3	(0.4)	4.2	(0.7)	1.8	(0.4)
**14–18 (n = 814)**									
	All	12.8	(1.1)**	31.5	(1.2)††	39.5	(1.5)	16.2	(1.1)
	Store	9.5	(1.0)	16.6	(1.2)	25.4	(1.5)	12.9	(0.9)
	Restaurants with fast food/Pizza	1.0	(0.3)	5.9	(0.8)	6.9	(0.9)	1.6	(0.3)
	Restaurant with waitstaff	—		1.0	(0.3)	3.5	(0.4)	—	
	School cafeteria	1.0	(0.3)	5.9	(1.8)	—		—	
	Other	—		2.1	(0.5)	3.3	(0.9)	1.5	(0.2)

* The proportion (%) of sodium consumed is defined as the sum of the amount of sodium consumed from each specific food category for all participants divided by the sum of sodium consumed from all food categories for all participants multiplied by 100. All estimates use 24-hour dietary recall, take into account the complex sampling design, and use dietary day 1 sample weights to account for nonresponse and weekend/weekday recalls.

† Eating occasions were defined by the participant. Responses were categorized as follows: breakfast was defined as “breakfast,” “desayuno,” or“almuerzo”; lunch was defined as “brunch,” “lunch,” or “comida”; dinner was defined as “dinner,” “supper,” or “cena”; and snack as “snack,” “drink,” “extended consumption (items that were consumed over a long period of time),” “merienda,” “entre comidas,” “botana,” “bocadillo,” “tentempie,” or “bebida.”

§ Place obtained was analyzed from responses to the question, “Where did you get this (most of the ingredients for this) [food name]?” Sources other than those shown were combined under “other.” The most common “Other” source listed was “from someone else/gift” (4.9%). Other sources each contributed <2% of sodium intake, including “missing,” “do not know,” and “other/specify” (<1%).

¶ Estimate not reported, either <1% or statistically unreliable, relative standard error ≥30%.

** Statistically significant difference in population proportion compared with children aged 6–10 years and children aged 11–13 years, by t-tests, p<0.05.

†† Statistically significant difference in population proportion compared with children aged 11–13 years, by t-tests, p<0.001.

## Footnotes

* Additional information available for Nutrition and Weight Status Objective 19 at http://www.healthypeople.gov/2020/topicsobjectives2020/objectiveslist.aspx?topicId=29.

† Additional information available at http://www.nel.gov/conclusion.cfm?conclusion_statement_id=250164&highlight=sodium&home=1.

§ According to the 2010 *Dietary Guidelines for Americans*, children aged ≥2 years should reduce their daily sodium intake to <2,300 mg and those who are African-American or have hypertension, diabetes, or chronic kidney disease should further reduce intake to 1,500 mg. Additional information is available at http://www.health.gov/dietaryguidelines/2010.asp. Using NHANES 2009–2010 data, 21.5% of U.S. children aged 6–18 years are African-American or have hypertension, diabetes or chronic kidney disease, and 99.2% of these children consume >1,500 mg sodium daily based on usual intakes. Of the remaining children, 87.9% consume ≥2,300 mg daily. Thus, 90.3% of U.S. children (0.215 × 99.2% + 0.785 × 87.9%) consume more sodium then recommended by the *Dietary Guidelines for Americans*. Tolerable upper intake levels are levels above which “usual intake may place an individual at risk of adverse effects from excessive nutrient intake.” The tolerable upper intake level for sodium intake for children aged 4–8 years is 1,900 mg/day, for children aged 9–13 years is 2,200 mg/day, and for children aged 14–18 years is 2,300 mg/day. Additional information available at http://www.iom.edu/reports/2004/dietary-reference-intakes-water-potassium-sodium-chloride-and-sulfate.aspx.

¶ Additional information regarding the sodium reduction targets is available at http://www.gpo.gov/fdsys/pkg/FR-2013-06-28/pdf/2013-15249.pdf and http://www.gpo.gov/fdsys/pkg/FR-2012-01-26/pdf/2012-1010.pdf. Note the baseline data for the sodium reduction targets were from the School Nutrition and Dietary Assessment Study III, the most current data available at the time of the study period. These data are available at http://www.fns.usda.gov/school-nutrition-dietary-assessment-study-iii.

** Additional information available at http://www.cdc.gov/nchs/nhanes.htm.

†† Additional information about the USDA Automated Multiple-Pass Method available at http://www.ars.usda.gov/services/docs.htm?docid=7710.

§§ Additional information available in the USDA Food and Nutrient Database for Dietary Studies, 5.0 at http://www.ars.usda.gov/services/docs.htm?docid=22370.

¶¶ Additional information available in the USDA National Nutrient Database for Standard Reference, Release 24 at http://www.ars.usda.gov/services/docs.htm?docid=22808.

*** Additional information available at http://www.ars.usda.gov/services/docs.htm?docid=23429.

††† Additional information available at http://www.cdc.gov/nccdphp/dnpa/growthcharts/resources/growthchart.pdf.

§§§ Sandwiches, like cheeseburgers, as identified by a single code in WWEIA.

¶¶¶ Mexican-mixed dishes, like burritos and tacos, as identified by a single code in WWEIA.

**** Additional information available at http://www.fns.usda.gov/sites/default/files/snda-iv_findings_0.pdf.

†††† Additional information available at http://www.cdc.gov/nchs/nhanes/nhanes2009-2010/dr1iff_f.htm.

§§§§ Additional information available at http://millionhearts.hhs.gov/index.html.
